# Plasma glial fibrillary acidic protein as a biomarker of disease progression in Parkinson’s disease: a prospective cohort study

**DOI:** 10.1186/s12916-023-03120-1

**Published:** 2023-11-06

**Authors:** Junyu Lin, Ruwei Ou, Chunyu Li, Yanbing Hou, Lingyu Zhang, Qianqian Wei, Dejiang Pang, Kuncheng Liu, Qirui Jiang, Tianmi Yang, Yi Xiao, Bi Zhao, Xueping Chen, Wei Song, Jing Yang, Ying Wu, Huifang Shang

**Affiliations:** Department of Neurology, West China Hospital, Sichuan University, Chengdu, 610041 Sichuan China

**Keywords:** Parkinson’s disease, Biomarkers, GFAP, Prospective cohort study, Disease progression

## Abstract

**Background:**

Reactive astrogliosis has been demonstrated to have a role in Parkinson’s disease (PD); however, astrocyte-specific plasma glial fibrillary acidic protein (GFAP)’s correlation with PD progression remains unknown. We aimed to determine whether plasma GFAP can monitor and predict PD progression.

**Methods:**

A total of 184 patients with PD and 95 healthy controls (HCs) were included in this prospective cohort study and followed-up for 5 years. Plasma GFAP, amyloid-beta (Aβ), p-tau181, and neurofilament light chain (NfL) were measured at baseline and at 1- and 2-year follow-ups. Motor and non-motor symptoms, activities of daily living, global cognitive function, executive function, and disease stage were evaluated using the Unified Parkinson’s Disease Rating Scale (UPDRS) part III, UPDRS-I, UPDRS-II, Montreal Cognitive Assessment (MoCA), Frontal Assessment Battery (FAB), and Hoehn and Yahr (H&Y) scales at each visit, respectively.

**Results:**

Plasma GFAP levels were higher in patients with PD (mean [SD]: 69.80 [36.18], pg/mL) compared to HCs (mean [SD]: 57.89 [23.54], pg/mL). Higher levels of GFAP were observed in female and older PD patients. The adjusted linear mixed-effects models showed that plasma GFAP levels were significantly associated with UPDRS-I scores (*β*: 0.006, 95% CI [0.001–0.011], *p* = 0.027). Higher baseline plasma GFAP correlated with faster increase in UPDRS-I (*β*: 0.237, 95% CI [0.055–0.419], *p* = 0.011) and UPDRS-III (*β*: 0.676, 95% CI [0.023–1.330], *p* = 0.043) scores and H&Y stage (*β*: 0.098, 95% CI [0.047–0.149], *p* < 0.001) and faster decrease in MoCA (*β*: − 0.501, 95% CI [− 0.768 to − 0.234], *p* < 0.001) and FAB scores (*β*: − 0.358, 95% CI [− 0.587 to − 0.129], *p* = 0.002). Higher baseline plasma GFAP predicted a more rapid progression to postural instability (hazard ratio: 1.009, 95% CI [1.001–1.017], *p* = 0.033).

**Conclusions:**

Plasma GFAP might be a potential biomarker for monitoring and predicting disease progression in PD.

**Supplementary Information:**

The online version contains supplementary material available at 10.1186/s12916-023-03120-1.

## Background

Glial fibrillary acidic protein (GFAP) is the main component of the astrocyte cytoskeleton, one of the most abundant cell types in the human central nervous system. Overexpression of GFAP is a critical marker for astrocytic activation. Research has highlighted the pivotal role of reactive astrogliosis in the pathogenesis of Parkinson’s disease (PD) [1, 2]. Studies have demonstrated that brain areas that are destroyed early in the development of PD have particularly high levels of GFAP and that there is an enteric glial reaction that leads to the overexpression of GFAP in PD patients [3, 4].

In neurological disorders involving astrocytic activation, astrocyte disintegration leads to the release of GFAP into the bloodstream, elevating plasma levels of GFAP. Therefore, plasma GFAP has been used as a diagnostic and prognostic biomarker for various diseases including traumatic brain injury (TBI) [5], cerebrovascular accidents [6], neuroinflammatory diseases [7], and, more recently, neurodegenerative disorders [8]. Elevated plasma GFAP has been reported to be a sensitive biomarker for tracking reactive astrogliosis and is positively correlated with amyloid-beta (Aβ) deposition in Alzheimer’s disease (AD) [9].

Although the initial link among PD and reactive astrogliosis has been established, few studies have investigated the role of plasma GFAP in PD. A previous study found elevated levels of GFAP in PD [10]. One study showed that plasma GFAP levels were higher in PD patients with dementia compared with PD patients without cognitive impairment or healthy controls (HCs) [11], whereas another study reported that ^11^C-BU99008, an imidazoline-2 binding site-specific positron emission tomography (PET) radioligand which can image reactive astrocytes in vivo, cannot differentiate patients with Parkinson’s disease dementia from HCs of similar age [12]. Therefore, the efficacy of plasma GFAP as a biomarker for monitoring disease severity and predicting disease progression in PD remains largely unknown.

We aimed to evaluate the evolution of plasma GFAP levels in a large prospective PD cohort and explore its correlation and potential predictive role with motor, non-motor, cognitive, and functional symptom progression in PD. In addition, we also included the neurofilament light chain (NfL) and the AD-related pathologies in the analyses given the reported close correlation between reactive astrogliosis, inflammatory responses, and neuro-axonal degeneration [13] and between plasma GFAP and AD-related pathologies [14].

## Methods

### Patient evaluation

The current study is part of an ongoing prospective longitudinal cohort study conducted at the Department of Neurology of West China Hospital of Sichuan University, which is designed to investigate the clinical and genetic characteristics and biomarkers of Chinese PD patients [15]. In the present study, early-stage PD patients (Hoehn and Yahr [H&Y] stage < 3) and age- and sex-matched HCs were included. Participants were recruited between February 2015 and November 2020 and followed for up to 5 years. The inclusion criteria included the following: [1] patients had a disease duration of less than 3 years at the time of enrollment; [2] the clinical picture as well as the brain magnetic resonance imaging (MRI) excluded vascular parkinsonism and atypical parkinsonism; [3] patients were willing to follow-up annually and were diagnosed with clinically established PD at the last follow-up according to the MDS clinical diagnostic criteria [16].

Demographic data, including sex, age, and years of education, were collected at baseline, as well as clinical data of PD patients (e.g., age at onset (age at first parkinsonian symptomatology), disease duration, and medication use). Detailed neurological examinations and face-to-face interviews were conducted by trained movement disorder specialists for all PD patients at baseline and every 12 months during the follow-up period in the clinically medication “OFF” state. Motor and non-motor symptoms and activities of daily living were evaluated using the Unified Parkinson’s Disease Rating Scale (UPDRS)-III, UPDRS-I, and UPDRS-II, respectively [17]. The H&Y scale was used to assess the disease stage [18]. Global cognitive and executive functioning were evaluated using the Chinese version of the Montreal Cognitive Assessment (MoCA) [19] and Frontal Assessment Battery (FAB) [20], respectively. Levodopa equivalent daily dose (LEDD) was calculated as previously reported [21].

### Standard protocol approvals, registrations, and patient consents

This study was approved by the Ethics Committee of West China Hospital of Sichuan University (No. 2015-236), and informed consent was obtained from all participants.

### Measurement of plasma GFAP and other biomarkers

Plasma levels of GFAP, Aβ40, Aβ42, phosphorylated tau-181 (p-tau181), and neurofilament light chain (NfL) were measured for all PD patients and HCs at baseline and re-measured for PD patients at the 1-year and 2-year follow-ups using an ultrasensitive single-molecule array (Simoa^TM^) technology (Quanterix, MA, US) on the automated Simoa^TM^ HD-X platform (GBIO, Hangzhou, China). The Neurology 4-Plex E Assay Kit (Cat No:103670) and p-tau181 Advantage V2 Assay Kit (Cat No:103714) were used. Plasma samples were diluted at a 1:4 ratio. Calibrators, quality controls, and all samples were measured in duplicate. The mean coefficients of variation (CVs) of duplicate measurements for concentration were 4.58% (GFAP), 1.71% (Ab40), 2.40% (Ab42), 7.22% (p-Tau181), and 4.45% (NfL). Samples with intra-assay CVs > 20% were re-measured. Assays were performed using kits with the same lot number. Operators were unaware of the disease status of the participants. The Aβ42/Aβ40 ratio was also calculated

### Genetic analysis

DNA was available for 159 of the PD patients. Two single nucleotide polymorphisms, rs429358 and rs7412, were used to detect the ε4 alleles of Apolipoprotein E (*APOE*), as previously reported [22].

### Statistical analyses

All statistical analyses were conducted using the R version 4.1.2. Statistical significance was defined as a two-tailed *p*-value < 0.05. Shapiro-Wilk analyses were conducted to test the normality of the variables. Continuous variables that were normally distributed are shown as means and standard deviations (SDs), and continuous variables that were not normally distributed are shown as medians and interquartile ranges (IQRs), whereas categorical variables are shown as numbers and percentages.

First, plasma GFAP levels were compared between HCs and PD patients at baseline using the Mann–Whitney *U* test. Receiver operating characteristic (ROC) curve analyses were performed to assess the ability of plasma GFAP to distinguish PD patients from HCs. Friedman tests were conducted to explore the longitudinal changes in plasma GFAP levels over 2 years in PD patients. Spearman correlation analyses were then conducted to assess the association between plasma GFAP levels and age in HCs and PD patients. Plasma GFAP levels were compared between males and females in HCs and PD patients at each visit and between *APOE*-ε4 carriers and noncarriers in PD patients at each visit using Mann–Whitney *U* tests. We conducted spearman correlation analyses between plasma GFAP levels and other plasma biomarkers, including Aβ40, Aβ42, p-tau181, NfL, and Aβ42/Aβ40 ratio in PD at each visit, as well as linear mixed-effects models between plasma GFAP levels and other plasma biomarkers over time. The plasma levels of GFAP over the 2-year follow-up period were also compared between high and low groups categorized by baseline plasma levels of Aβ40, Aβ42, p-tau181, and NfL and Aβ42/Aβ40 ratio using the linear mixed-effects models. The highest and lowest tertiles were used to dichotomize the biomarker levels to make a better distinguishing from pure PD with concurrent AD pathology. Patients with the highest tertile of baseline biomarker were classified into the high biomarker group, while patients with the lowest tertile of the baseline biomarker were classified into the low biomarker group. The GFAP level was used as the dependent variable. The baseline plasma biomarkers, the follow-up time, age, sex, and the interaction term of time*biomarker were treated as fixed effects, and the intercept was treated as a random effect. FDR correction was conducted for multiple comparisons. The plasma levels of GFAP over the 2-year follow-up period were also compared between *APOE*-ε4 carriers and noncarriers using linear mixed-effects models. The GFAP level was used as the dependent variable. The *APOE* status, the follow-up time, age, sex, and the interaction term of time*APOE status were treated as fixed effects, and the intercept was treated as a random effect.

Second, correlations between plasma GFAP and motor, non-motor, cognitive, and functional performance over the 2-year follow-up were investigated using linear mixed-effects models, accounting for correlations among repeated measures and variable length of follow-up. Repeated measures of GFAP were treated as a fixed effect, and the intercept was treated as a random effect. The time-varying scores of UPDRS-III, UPDRS-I, UPDRS-II, MoCA, FAB, and H&Y stage were used as the dependent variables in each unadjusted model. Adjusted models also included sex, age at baseline, disease duration at baseline, follow-up time, and education (only for cognitive) as covariates. We also compared the clinical progress between patients who had a downward or upward trends of GFAP over time using repetitive measurement deviation analyses.

Third, linear mixed-effects models were applied to explore whether baseline plasma GFAP levels could predict motor, non-motor, cognitive, and functional progression in PD. Given that the H&Y stage is an ordinal variable, the generalized linear mixed effect models were used for analyzing the H&Y stage. The predictive ability of baseline GFAP on disease progression was examined through the interactions of baseline GFAP with follow-up time. The baseline plasma GFAP was converted into a binary variable in the analyses. Patients with the highest tertile (> 76.42 pg/mL) of baseline GFAP were classified into the high GFAP group, while patients with the lowest tertile (≤ 48.36 pg/mL) of the baseline GFAP were classified into the low GFAP group. Baseline GFAP, follow-up time, and their interaction were treated as a fixed effect, and the intercept was treated as a random effect. The time-varying scores of UPDRS-III, UPDRS-I, UPDRS-II, MoCA, FAB, and H&Y stage were used as the dependent variables, and sex, age at baseline, disease duration at baseline, and baseline scores of UPDRS-III, UPDRS-I, UPDRS-II, MoCA, FAB, and H&Y stage were included as covariates. Sensitivity analyses were conducted by adding other biomarkers as covariates in the models.

Finally, the Kaplan-Meier survival analyses were performed using postural instability (defined as H&Y stage ≥ 3) as the outcome event, and the log-rank tests were conducted to compare the progression to postural instability between the high and low plasma GFAP groups. Univariate and multivariate Cox proportional hazards regression with sex, age, disease duration, baseline UPDRS-III score, baseline MoCA scores, and baseline plasma GFAP levels as predictors were performed to explore whether the baseline plasma GFAP level could predict the progression to postural instability. ROC curve analyses were performed to assess the ability of plasma GFAP to distinguish the patients who would progress to postural instability within 5 years.

## Results

### Participants

A total of 184 PD patients (99 males [53.8%], mean age: 57.60 ± 11.10 years, disease duration: 1.52 ± 0.86 years, mean follow-up time: 4.02 years) and 95 HCs (49 males [51.6%], age: 55.07 ± 7.31 years) were included at baseline. For neurological examinations and face-to-face interviews, 184 (100%) patients finished the 1-year follow-up, 172 (93.5%) patients finished the 2-year follow-up (one died, one was lost to follow-up, and 10 did not reach the time for the second year’s visit), 117 (63.6%) patients finished the 3-year follow-up (two died, five were lost to follow-up, and 60 did not reach the time for the third year’s visit), 60 (32.6%) patients finished the 4-year follow-up (four died, eight were lost to follow-up, and 112 did not reach the time for the fourth year’s visit), and 22 (12.0%) patients finished the 5-year follow-up (six died, 15 were lost to follow-up, and 141 did not reach the time for the fifth year’s visit; Table 1). For the measurement of plasma biomarkers, 184 (100%) and 124 (67.4%) patients were assessed at baseline and 1-year follow-up and 2-year follow-up, respectively. In total, 739 clinical measurements and 492 biomarkers measurements were analyzed in this study.

**Table 1:** Demographic and clinical features of the recruited patients with PD

	Baseline (*n* = 184)	1 year (*n* = 184)	2 years (*n* = 172)	3 years (*n* = 117)	4 years (*n* = 60)	5 years (*n* = 22)	HC (*n* = 95)
Age, years, mean (SD)	57.79 (11.18)	58.89 (11.21)	60.10 (11.39)	61.78 (10.42)	63.10 (11.09)	66.47 (8.54)	55.07 (7.31)
Male sex, *n* (%)	99 (53.80)	99 (53.80)	92 (53.49)	65 (55.56)	29 (48.33)	9 (40.90)	49 (51.6%)
Disease duration, mean (SD)	1.98 (1.20)	3.08 (1.24)	4.13 (1.28)	5.12 (1.18)	6.16 (1.16)	6.77 (1.19)	/
Education, years, mean (SD)	10.45 (3.88)	10.45 (3.88)	10.36 (3.86)	10.06 (3.86)	10.05 (4.51)	9.96 (4.42)	/
Levodopa use, *n* (%)	98 (53.26)	132 (71.74)	137 (79.65)	101 (86.32)	53 (88.33)	19 (86.40)	/
Dopaminergic agonist use, *n* (%)	65 (35.33)	125 (67.93)	136 (79.07)	99 (84.62)	54 (90.00)	18 (81.80)	/
LEDD, mg/day, mean (SD)	235.51 (249.59)	375.80 (213.14)	447.28 (233.58)	522.06 (238.61)	537.49 (276.06)	542.04 (243.59)	/
UPDRS-I score, mean (SD)	0.99 (1.59)	1.17 (1.50)	1.53 (1.62)	1.77 (1.94)	1.88 (1.99)	2.41 (2.58)	/
UPDRS-II score, mean (SD)	5.91 (4.18)	7.40 (4.29)	8.13 (4.84)	10.43 (5.23)	11.27 (4.05)	13.09 (7.71)	/
UPDRS-III score, mean (SD)	22.90 (8.70)	26.85 (9.09)	30.41 (9.45)	34.26 (10.40)	37.33 (9.46)	40.55 (15.40)	/
H&Y, mean (SD)	1.89 (0.33)	2.04 (0.30)	2.16 (0.44)	2.22 (0.46)	2.26 (0.46)	2.39 (0.77)	/
MoCA score, mean (SD)	25.46 (3.51)	24.87 (3.66)	24.49 (4.06)	24.11 (4.79)	23.33 (4.76)	22.36 (5.82)	26.17 (2.88)
FAB score, mean (SD)	16.10 (2.23)	16.12 (2.11)	15.70 (2.41)	15.48 (2.76)	14.52 (3.17)	14.50 (3.69)	/
NfL, median (IQR), pg/ml	9.25 (6.30)	9.70 (6.69)	9.82 (7.94)	/	/	/	6.938 (4.404)
Aβ40, median (IQR), pg/ml	93.27 (17.98)	91.97 (19.68)	90.44 (20.91)	/	/	/	88.863 (17.979)
Aβ42, median (IQR), pg/ml	7.45 (1.85)	7.48 (2.07)	6.91 (1.72)	/	/	/	6.965 (1.962)
P-tau181, median (IQR), pg/ml	1.47 (0.70)	1.53 (0.87)	1.64 (0.89)	/	/	/	1.364 (0.623)

*Abbreviations*: *PD* Parkinson’s disease, *LEDD* Levodopa equivalent daily dose, *UPDRS-I* Unified Parkinson’s Disease Rating Scale part I, *UPDRS-II* Unified Parkinson’s Disease Rating Scale part II, *UPDRS-III* Unified Parkinson’s Disease Rating Scale part III, *H&Y* Hoehn and Yahr, *MoCA* Montreal Cognitive Assessment, *FAB* Frontal assessment battery, *NfL* Neurofilament light chain, *Aβ* Amyloid-beta, *p-tau181* Phosphorylated tau-181

### Evolution and comparison of plasma GFAP

**Fig. 1:**
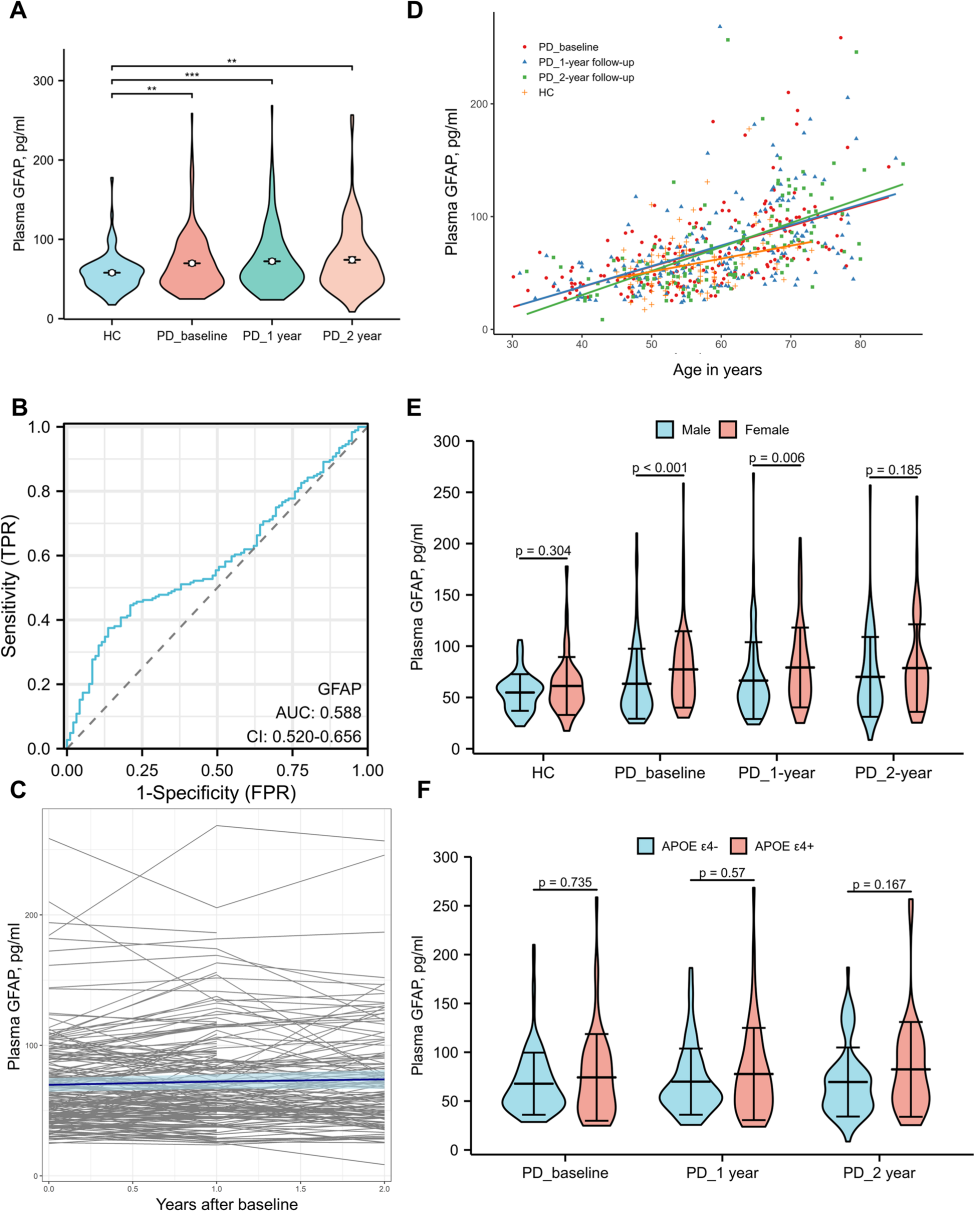
Evolution and comparison of plasma GFAP in PD patients. **A** Comparison of the plasma GFAP levels between HCs and PD patients. Significance was examined using the Mann–Whitney *U* test. **B** Receiver operating characteristic (ROC) curve of the plasma GFAP levels on distinguishing PD patients from HCs. **C** Evolution of the plasma GFAP levels in PD over 2 years. **D** Correlation between plasma GFAP levels and age in HCs and PD patients at baseline and 1- and 2-year follow-ups. **E** Comparison of plasma GFAP levels between males and females in HCs and PD patients at baseline and 1- and 2-year follow-ups. Significance was determined using the Mann–Whitney *U* test. **F** Comparison of plasma GFAP levels between *APOE*-ε4 carriers and *APOE*-ε4 non-carriers in PD patients at baseline and 1- and 2-year follow-ups. Significance was examined using the Mann–Whitney *U* test

**Fig. 2:**
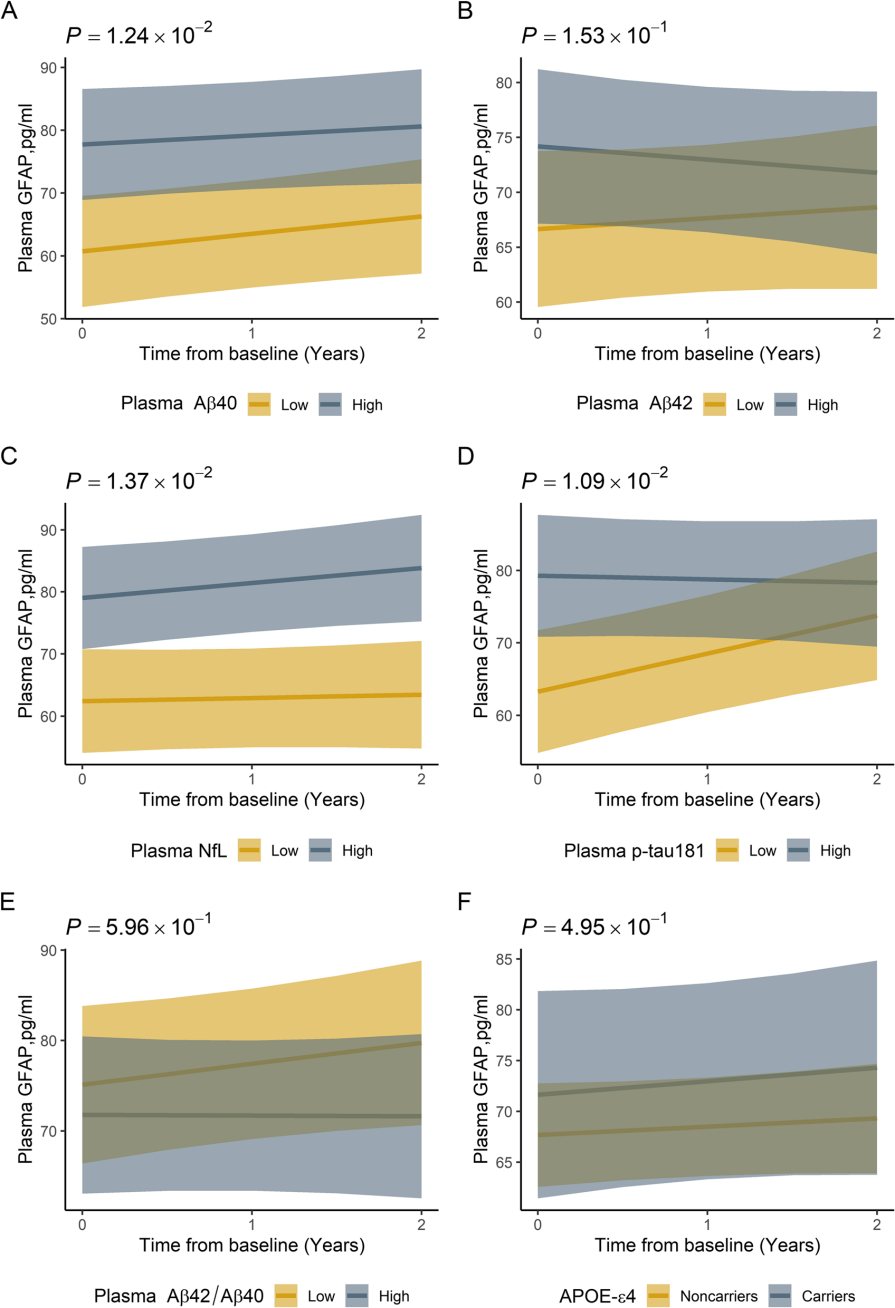
Change over time of the plasma GFAP between groups. **A** Change over time of the plasma GFAP levels for Parkinson’s disease patients with high baseline plasma Aβ40 compared to those with low baseline plasma Aβ40. **B** Change over time of the plasma GFAP levels for Parkinson’s disease patients with high baseline plasma Aβ42 compared to those with low baseline plasma Aβ42. **C** Change over time of the plasma GFAP levels for Parkinson’s disease patients with high baseline plasma NfL compared to those with low baseline plasma NfL. **D** Change over time of the plasma GFAP levels for Parkinson’s disease patients with high baseline plasma p-tau181 compared to those with low baseline plasma p-tau181. **E** Change over time of the plasma GFAP levels for Parkinson’s disease patients with high baseline plasma Aβ42/Aβ40 compared to those with low baseline plasma Aβ42/Aβ40. **F** Change over time of the plasma GFAP levels for APOE-ε4 carriers and APOE-ε4 non-carriers. Data shown are the mean predicted plasma GFAP levels (± 1SE) based on output from a linear mixed-effects model, adjusted for sex and age at baseline. *p* value for comparison between groups

Plasma GFAP levels were significantly higher in patients with PD at baseline (mean [SD]: 69.80 [36.18], pg/mL) compared to HCs (mean [SD]: 57.89 [23.54], pg/mL; Fig. 1A and Additional file 1: Table S1). The ROC analyses showed that the plasma GFAP level had a low accuracy to differentiate PD patients from HCs (area under the curve (AUC): 0.588, 95% CI [0.520 to 0.656]) (Fig. 1B). The Friedman test showed that plasma GFAP levels in PD increased significantly (*p* < 0.001) over the 2-year follow-up (baseline: mean [SD]: 69.80 [36.18] pg/mL, 2-year follow-up: mean [SD]: 74.12 [40.79] pg/mL; Fig. 1A, C, and Additional file 1: Table S1). Plasma GFAP levels were positively correlated with age in HCs (*r* = 0.319, *p* = 0.002) and PD patients at baseline (*r* = 0.628, *p* < 0.001) and 1-year (*r* = 0.602, *p* < 0.001) and 2-year (*r* = 0.665, *p* < 0.001) follow-ups (Fig. 1D). Plasma GFAP levels were significantly higher in women than men in PD patients at baseline and the 1-year follow-up, while comparable in HCs and PD patients at the 2-year follow-up (Fig. 1E). Plasma GFAP levels were not significantly different between *APOE*-ε4 carriers and noncarriers in PD patients at baseline or 1- and 2-year follow-up (Figs. 1F and 2F).

### Correlation between plasma GFAP and other biomarkers

Plasma GFAP levels were positively correlated with plasma levels of Aβ40, Aβ42, p-tau181, and NfL at every visit (Additional file 1: Table S2) as well as over time (Additional file 1: Table S3). The linear mixed-effects models showed that after age, sex, and follow-up time were adjusted, patients with high baseline plasma Aβ40 (*β* = 17.013, 95% CI [3.852 to 30.174], *p* = 0.012), NfL (*β* = 16.601, 95% CI [3.565 to 29.637], *p* = 0.014), or p-tau 181 (*β* = 15.991, 95% CI [3.835 to 28.147], *p* = 0.001) levels had consistently higher levels of plasma GFAP over long-term follow-up than those with low baseline plasma Aβ40, NfL, or p-tau 181 levels (Fig. 2).

### Correlation between plasma GFAP and motor, non-motor, cognitive, and functional scores

The adjusted linear mixed-effects models showed that plasma GFAP levels were significantly associated with UPDRS-I scores over the 2-year follow-up after adjusting for sex, age at baseline, disease duration at baseline, and follow-up time (*β*: 0.006, 95% CI [0.001 to 0.011], *p* = 0.027, Table 2). Among the 184 included patients with PD, 85 patients had downward trends of GFAP, while 99 patients had upward trends of GFAP (Additional file 1: Table S4). We have drawn graphics to show the clinical process of the patients with upward or downward trends of GFAP (Additional file 1: Fig. S1). As shown in Additional file 1: Fig. S1, PD patients with a downward or flat trends of GFAP had lower scores of UPDRS-I, UPDRS-II, and UPDRS-III, higher scores of MOCA and FAB, and lower H&Y stage. The results suggested a consistence of the change of plasma GFAP and clinical process in PD.

**Table 2:** Correlation between plasma GFAP and motor, non-motor, cognitive, and functional scores in PD over time

Variable	Adjusted model	*P* value
	*β* (95% CI)	
UPDRS-I	0.006 (0.001 ~ 0.011)^a^	0.027*
UPDRS-II	0.002 (− 0.012 ~ 0.016)^a^	0.792
UPDRS-III	0.012 (− 0.006 ~ 0.031)^a^	0.198
H&Y	0.001 (− 0.0004 ~ 0.002)^a^	0.230
MOCA	− 0.004 (− 0.014~ 0.006)^b^	0.430
FAB	− 0.001 (− 0.009 ~ 0.006)^b^	0.714

*Abbreviations*: *GFAP* Glial fibrillary acidic protein, *PD* Parkinson’s disease, *UPDRS-I* Unified Parkinson’s Disease Rating Scale part I, *UPDRS-II*, Unified Parkinson’s Disease Rating Scale part II; *UPDRS-III* Unified Parkinson’s Disease Rating Scale part III, *H&Y* Hoehn and Yahr; *MoCA* Montreal Cognitive Assessment, *FAB* Frontal assessment battery

^*^Significant based on linear mixed-effects models

^a^Covariates: age at baseline, sex, time, and duration at baseline

^b^Covariates: age at baseline, sex, time, duration at baseline, and education

### Prediction of motor, non-motor, cognitive, and functional progression using baseline plasma GFAP

**Fig. 3:**
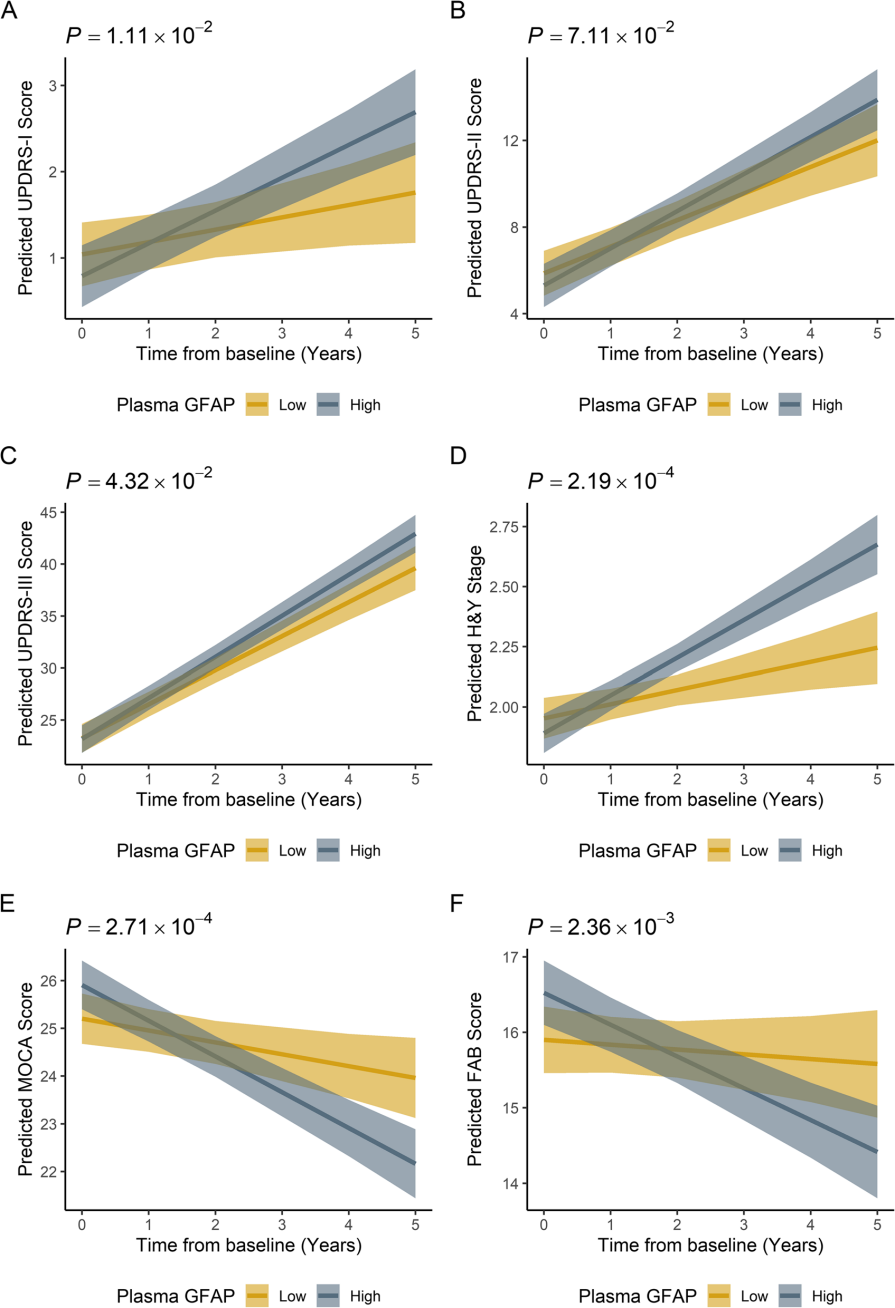
Relationship between baseline plasma GFAP with longitudinal changes of motor, non-motor, cognitive, and functional scores. Data shown are the mean predicted scores of UPDRS-I, UPDRS-II, UPDRS-III, H&Y stage, MoCA, and FAB (± 1 SE) based on output from a linear mixed-effects models or generalized linear mixed effect models (only for H&Y stage), adjusted for sex, age at baseline, disease duration at baseline, and baseline scores of UPDRS-I, UPDRS-II, UPDRS-III, H&Y stage, MoCA, and FAB. *P* value depicts group*time interaction. **A** Change over time of the UPDRS-I score for PD patients with high baseline plasma GFAP compared to those with low baseline plasma GFAP. **B** Change over time of the UPDRS-II score for PD patients with high baseline plasma GFAP compared to those with low baseline plasma GFAP. **C** Change over time of the UPDRS-III score for PD patients with high baseline plasma GFAP compared to those with low baseline plasma GFAP. **D** Change over time of the H&Y stage for PD patients with high baseline plasma GFAP compared to those with low baseline plasma GFAP. **E** Change over time of the MoCA score for PD patients with high baseline plasma GFAP compared to those with low baseline plasma GFAP. **F** Change over time of the FAB score for PD patients with high baseline plasma GFAP compared to those with low baseline plasma GFAP

Linear mixed-effects models showed that baseline plasma GFAP could predict long-term motor, non-motor, and cognitive progression in PD patients. PD patients with a high baseline plasma GFAP (> 76.42 pg/mL) showed a more rapid increase in UPDRS-I (*β*: 0.237, 95% CI [0.055 to 0.419], *p* = 0.011) and UPDRS-III scores (*β*: 0.676, 95% CI [0.023 to 1.330], *p* = 0.043) and H&Y stage (*β*: 0.098, 95% CI [0.047 to 0.149], *p* < 0.001) than those with a low baseline plasma GFAP (≤ 48.36 pg/mL; Fig. 3A–D). PD patients with high baseline plasma GFAP also showed a more rapid decline in MoCA scores (*β*: -0.501, 95% CI [− 0.768 to − 0.234], *p* < 0.001) and FAB scores (*β*: − 0.358, 95% CI [− 0.587 to − 0.129], *p* = 0.002) than those with low baseline plasma GFAP (≤ 48.36 pg/mL; Fig. 3E–F). These results remained stable in the sensitivity analyses (Table 3).

**Table 3:** Relationship between baseline plasma GFAP with longitudinal motor, non-motor, cognitive, and functional deterioration with other biomarkers adjusted

	Change in UPDRS-I score		Change in UPDRS-II score		Change in UPDRS-III score	
Adjusted biomarkers	*β* (95% CI)	*p* value	*β* (95% CI)	*p* value	*β* (95% CI)	*p* value
With plasma Aβ40 adjusted	0.237 (0.055 ~ 0.419)	0.011*	0.484 (− 0.043 ~ 1.012)	0.073	0.678 (0.024 ~ 1.331)	0.043*
With plasma Aβ42 adjusted	0.236 (0.054 ~ 0.418)	0.012*	0.486 (− 0.041 ~ 1.013)	0.072	0.673 (0.019 ~ 1.327)	0.044*
With plasma NfL adjusted	0.238 (0.056 ~ 0.420)	0.011*	0.493 (− 0.034 ~ 1.020)	0.067	0.684 (0.030 ~ 1.337)	0.041*
With plasma p-tau181 adjusted	0.233 (0.051 ~ 0.415)	0.013*	0.486 (− 0.042 ~ 1.013)	0.072	0.665 (0.010 ~ 1.319)	0.047*
With plasma Aβ42/Aβ40 adjusted	0.233 (0.051 ~ 0.415)	0.013*	0.477 (− 0.051 ~ 1.006)	0.077	0.662 (0.008 ~ 1.317)	0.048*
	Change in H&Y stage		Change in MoCA score		Change in FAB score	
Adjusted biomarkers	*β* (95% CI)	*p* value	*β* (95% CI)	*p* value	*β* (95% CI)	*p* value
With plasma A*β*40 adjusted	0.191 (0.105 ~ 0.277)	< 0.001*	− 0.499 (− 0.766 ~ − 0.231)	< 0.001*	− 0.358 (− 0.588 ~ − 0.129)	0.002*
With plasma Aβ42 adjusted	0.146 (0.066 ~ 0.226)	< 0.001*	− 0.502 (− 0.769 ~ − 0.234)	< 0.001*	− 0.357 (− 0.587 ~ − 0.128)	0.002*
With plasma NfL adjusted	0.141 (0.060 ~ 0.221)	< 0.001*	− 0.503 (− 0.771 ~ − 0.236)	< 0.001*	− 0.362 (− 0.591 ~ − 0.132)	0.002*
With plasma p-tau181 adjusted	0.157 (0.075 ~ 0.239)	< 0.001*	− 0.487 (− 0.765 ~ − 0.230)	< 0.001*	− 0.352 (− 0.581 ~ − 0.123)	0.003*
With plasma Aβ42/Aβ40 adjusted	0.154 (0.072 ~ 0.236)	< 0.001*	− 0.499 (− 0.767 ~ − 0.231)	< 0.001*	− 0.353 (− 0.583 ~ − 0.123)	0.003*

*Abbreviations*: *GFAP* Glial fibrillary acidic protein, *UPDRS-I* Unified Parkinson’s Disease Rating Scale part I, *UPDRS-II* Unified Parkinson’s Disease Rating Scale part II, *UPDRS-III* Unified Parkinson’s Disease Rating Scale part III, *H&Y* Hoehn and Yahr, *MoCA* Montreal Cognitive Assessment, *FAB* Frontal assessment battery, *NfL* Neurofilament light chain, *Aβ* amyloid-beta, *p-tau181* Phosphorylated tau-181

^*^Significant based on linear mixed-effects models and generalized linear mixed effect models (only for H&Y stage). *p* value for GFAP group*time interaction

### Prediction of baseline plasma GFAP on progression to postural instability

**Fig. 4:**
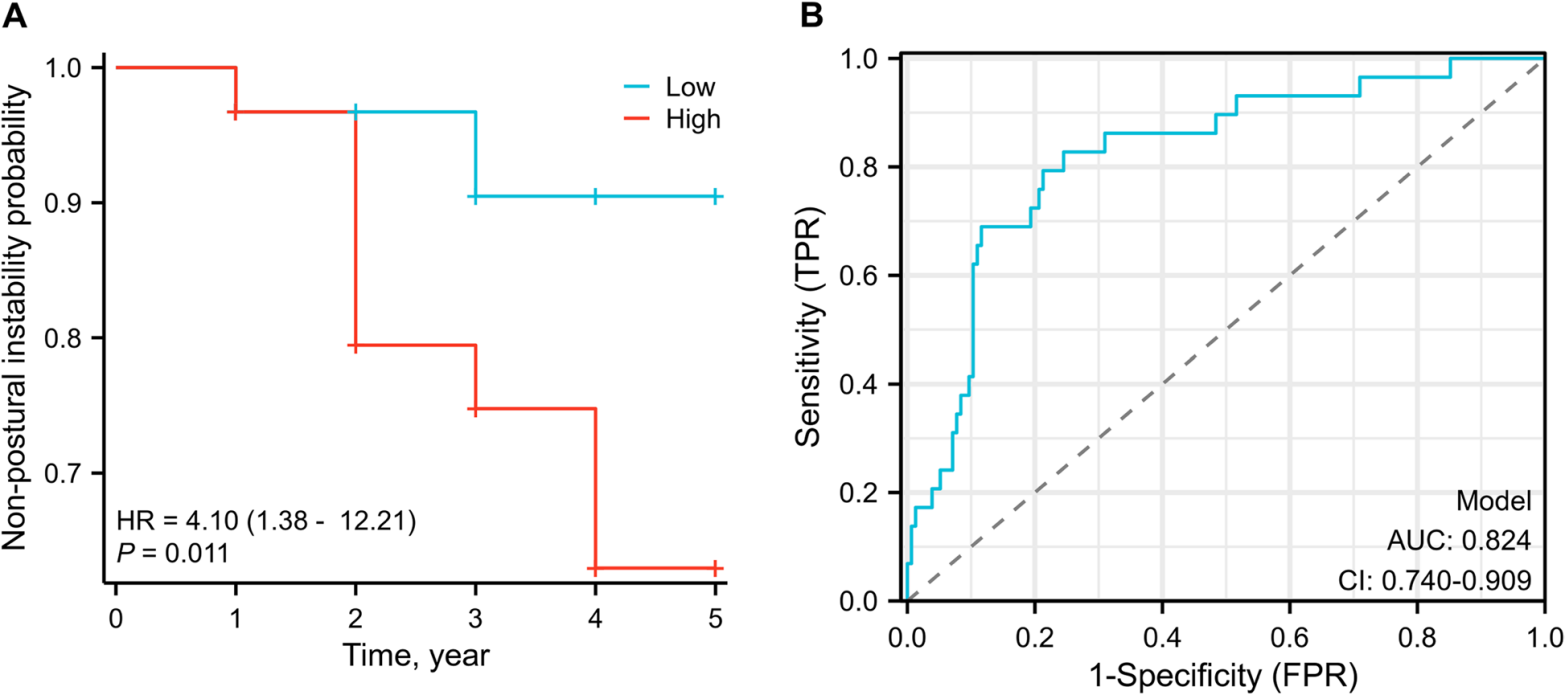
Plasma GFAP predicts progression to postural instability in PD. **A** Kaplan–Meir survival curves for progress to postural instability by plasma GFAP tertiles. **B** ROC curves of the plasma GFAP level combined with sex and baseline UPDRS-III score for distinguishing patients who would progress to postural instability within 5 years

We explored if baseline plasma GFAP could predict progression to postural instability using the Kaplan–Meir survival analysis and Cox proportional hazards regression models. Of the 184 PD patients, 29 developed postural instability during follow-up. Kaplan–Meir survival curves and the log-rank tests showed that patients with the highest tertile of baseline GFAP had a more rapid progression to postural instability compared to those with the lowest tertile (*p* = 0.010; Fig. 4A). After age, sex, disease duration, and baseline MoCA scores were adjusted, higher baseline plasma GFAP levels (HR: 1.009, 95% CI [1.001 to 1.017], *p* = 0.033) and higher baseline UPDRS-III scores (HR: 1.107, 95% CI [1.063 to 1.153], *p* < 0.001) predicted a more rapid progression to postural instability in the multivariate Cox regression models (Additional file 1: Table S5). ROC analyses showed that a combination of baseline plasma GFAP levels and baseline UPDRS-III scores had a high accuracy (AUC: 0.824, 95% CI [0.740 to 0.909]) for distinguishing patients who would progress to postural instability within 5 years (Fig. 4B).

## Discussion

By conducting a comprehensive analysis of plasma GFAP in a large prospective cohort of PD patients, we obtained several important findings. First, the plasma GFAP levels increased with disease duration in PD, and higher levels of plasma GFAP were observed in patients with PD compared with HCs. Second, plasma GFAP levels increased with age in all participants, and women demonstrated higher levels of plasma GFAP than men. Third, plasma GFAP levels were positively correlated with other plasma biomarkers, especially the NfL. Finally, plasma GFAP can be used as a potential biomarker to monitor and predict the motor, non-motor, and cognitive progression of PD.

In agreement with our findings, previous studies have detected an elevated level of plasma GFAP in PD [10] and other neurodegenerative disorders such as AD [23], Lewy body dementia [11], and frontotemporal dementia [24], as well as other neurological disorders such as traumatic brain injury (TBI) [5], cerebrovascular accidents [6], and neuroinflammatory diseases [7]. Other astrocytes specific markers, such as S100B, have been found to be elevated in PD [25]. Widely distributed in the central nervous system, astrocytes are essential for the normal function of synapses and axonal metabolic maintenance and are important in the formation of the blood-brain barrier. As an astrocyte-specific protein, plasma GFAP is a promising and easily available blood biomarker for reactive astrogliosis. For the first time, increases in plasma GFAP levels were observed in our current PD cohort along with disease progression; this is consistent with previous studies on patients with mild cognitive impairment (MCI) [26] and preclinical AD [27]. Like the current study, previous studies have found a positive association between age and plasma GFAP levels in patients with AD [9] and frontotemporal dementia (FTD) [28] and higher plasma GFAP levels in females compared to males were observed in patients with AD [9] and TBI [29]. A recent study found a large sex effect for neurite dispersion by observing a negative association between GFAP and neurite dispersion only in females, indicating that sex is an important modifier of the complex associations between astrogliosis, immune dysregulation, and brain microstructure [30]. These results indicated that plasma GFAP is a sensitive potential biomarker for tracking the reactive astrogliosis in PD and other neurodegenerative disorders. However, given the overlap of GFAP values between PD patients and HCs, caution should be exercised when recommending individual use.

The most important finding of our current study was the correlation between plasma GFAP levels and motor, non-motor, and cognitive severity in PD, as well as the correlation between the change of GFAP and the clinical progress. In addition, a predictive role of baseline plasma GFAP was identified on motor, non-motor, and cognitive symptoms progression, and postural instability in PD. Postural instability is a major milestone and the best index of disease progression in PD, as it was reported to evolve more rapidly than other motor features [31]. Growing evidence suggests a correlation between GFAP and PD pathology [1, 32–34]. Reportedly, brain areas that are destroyed early in the development of PD have particularly high levels of GFAP [3]. An animal study found the injection of Adeno-GFAP-GFP virus into the substantia nigra pars compacta caused severe reactive astrogliosis and exacerbated the accumulation of α-synuclein [1]. An animal study found that reactive astrocytes induced excess inflammatory responses, leading to neuronal degeneration in mouse models of PD [35]. Another study assessed astrogliosis in PD using 11C-BU99008 PET and found reactive astroglia in the early stages of PD, reflecting potential neuroprotective compensatory mechanisms and pro-inflammatory upregulation in response to α-synuclein accumulation [33]. A study detected that neuronal α-synuclein will transmit to astroglia, leading to inflammatory responses [13]. These results supported the hypothesis that α-synuclein accumulation induces the reactive astrocytes, and then reactive astrocytes induce excess inflammatory responses and ultimately the neuronal degeneration.

Several studies have reported a correlation between GFAP and cognitive performance in PD and other neurodegenerative diseases. One study revealed increased levels of cerebrospinal fluid (CSF) GFAP in PD-MCI patients compared to cognitively unimpaired patients [36]. One study detected elevated GFAP levels in PD patients with dementia compared with those without cognitive impairment or HCs [11], whereas another study found that ^11^C-BU99008 PET cannot differentiate patients with Parkinson’s disease dementia from HCs [12]. One study found that plasma GFAP levels were significantly associated with cognitive performance in a cohort including individuals with subjective cognitive decline, MCI, and AD [37]. Other studies have identified significant correlations between plasma GFAP level and cognition in patients with preclinical AD [27] and FTD [24, 28]. These results as well as our findings suggested the monitoring potential of plasma GFAP on cognitive performance in neurodegenerative diseases [27]. For the predictive role on cognitive performance detected in our study, a possible explanation is the strong relationship between plasma GFAP and AD-related pathology [14], which was also supported by the correlation analyses in the current study. In patients with preclinical AD, plasma GFAP levels were found to be higher in cognitively unimpaired older adults with the presence of brain amyloidosis compared with those without [27, 38], suggesting that plasma GFAP is an early biomarker for Aβ pathology. A similar predictive role of plasma GFAP on cognitive decline has also been reported in patients with AD [39, 40] and MCI [26]. Although the presence of AD-related pathology in PD remains to be a controversy, some studies supported a role of AD-related pathology in the development of the cognitive decline in PD [41], especially the Aβ pathology [42]. However, the results from another study suggested not AD-related pathology, but Lewy body pathology contributed to dementia in PD [43].

In the current study, we found significant correlations between plasma GFAP and NfL in PD. In particular, consistently higher levels of plasma GFAP were found in PD patients with higher baseline levels of NfL. As a reliable biomarker for neuronal injury and axonal degeneration, plasma NfL has been widely reported to be correlated with disease severity and progression in PD and other neurodegenerative disorders [44, 45]. Baseline plasma NfL levels were also able to predict cognitive decline [46], psychotic symptoms [47], and non-motor symptoms [48] in PD. Strong correlations between plasma GFAP and NfL have been detected in previous studies of neurodegenerative disorders, such as AD [9] and FTD [28], suggesting a concomitance and possible interplay of axonal and astrocytic dysfunction in the development of neurodegenerative disorders. Sensitivity analyses showed that the predictive role of plasma GFAP remained unchanged after other biomarkers were adjusted, suggesting that plasma GFAP has a predictive role independent of other biomarkers. A recent study found that GFAP was associated with clinical AD incidence up to 17 years before diagnosis, while p-tau181 and NfL within 9 years in a community-based cohort, suggesting GFAP’s utility as an earlier biomarker than p-tau181 and NfL [49]. These results indicate that astrocytic activation may begin earlier, namely prior to symptom development and other biomarker aggregation in neurodegenerative disorders.

Our study is the first to comprehensively evaluate the role of plasma GFAP in PD using a prospective longitudinal cohort study and yields some important findings. We demonstrated the predictive role of plasma GFAP on disease progression in PD for the first time. Our findings suggest that reactive astrogliosis and related inflammation are associated with subsequent motor and non-motor symptom deterioration and cognitive decline in PD. Moreover, plasma GFAP is an accessible and reliable biomarker for monitoring disease severity and an early potential biomarker for predicting motor, non-motor, and cognitive progression in PD. Our study offers the evidence of the inclusion of GFAP in clinical trials testing potential disease modifying therapy and supports the need for further exploration of astrocytic pathology in PD and possibly targeting astrocytes as a potential therapeutic target. Future studies are needed to investigate whether plasma GFAP has a monitoring role also in patients at risk of PD, such as patients with RBD or relatives of patients with familial PD aggregation.

However, several limitations should be acknowledged. First, CSF was not obtained from participants; therefore, we did not conduct the comparison between plasma and CSF GFAP. However, plasma GFAP has been reported to be an even more sensitive biomarker than CSF GFAP [9]. Second, other astrocyte markers, such as S100B and YKL40, were not examined. Third, brain MRI cannot absolutely rule out atypical parkinsonism of all patients, especially in the early phases of parkinsonism syndromes. In addition, tau-PET or Aβ-PET were not performed, so the possibility of concurrent AD pathology in the PD patients could not be completely excluded. Forth, we used the old UPDRS scale instead of the more updated MDS-UPDRS and the correlation to PD medications are lacking. Fifth, the correlation coefficient revealed in the current study is relatively small, and the follow-up data of HCs are lacking. The long-term follow-up has a relatively large attrition rate, which might affect the results of the survival analyses. In addition, only postural instability was used as a milestone in our study. Studies with more complete data and larger sample sizes are required to confirm our results in the future. Finally, GFAP elevation is not specific for PD and is probably only useful in monitoring disease in PD patients without other neurological comorbidities, and mechanism researches for GFAP in PD are still needed.

## Conclusions

In conclusion, plasma GFAP levels were higher in PD compared with HCs and increased with disease progression. Higher levels of plasma GFAP were observed in older and female PD patients. Plasma GFAP can be used as an accessible biomarker to monitor motor, non-motor, and cognitive performance and as an early potential biomarker to predict longitudinal motor and non-motor symptoms deterioration, cognitive decline, and postural instability in PD. These findings emphasize the role of reactive astrogliosis in PD and suggest the great potential of plasma GFAP in neurodegenerative disorders.

### Supplementary Information

**Additional file 1:** **Table S1.** Plasma GFAP levels of the included participants. **Table S2.** Correlation of plasma GFAP with other biomarkers at each visit. **Table S3.** Correlation of plasma GFAP with other biomarkers over time. **Table S4.** Comparison of clinical scores between PD patients with upward or downward trends of GFAP. **Table S5**. Cox proportional hazards regression models .**Fig S1.** Comparison of clinical progress between PD patients with upward or downward trends of GFAP.

## Data Availability

The datasets used and/or analyzed during the current study available from the corresponding author on reasonable request.

## Abbreviations

PD: Parkinson’s disease
GFAP: Plasma glial fibrillary acidic protein
HCs: Healthy controls
Aβ: Amyloid-beta
p-tau181: Phosphorylated tau-181
NfL: Neurofilament light chain
UPDRS III: Unified Parkinson’s Disease Rating Scale part III
UPDRS I: Unified Parkinson’s Disease Rating Scale part I
UPDRS II: Unified Parkinson’s Disease Rating Scale part II
MoCA: Montreal Cognitive Assessment
FAB: Frontal Assessment Battery
H&Y: Hoehn and Yahr
AD: Alzheimer’s disease
LEDD: Levodopa equivalent daily dose
CVs: Coefficients of variation
Simoa: Single-molecule array
APOE: Apolipoprotein E
SDs: Standard deviations
IQRs: Inter-quartile ranges
HR: Hazard ratio
TBI: Traumatic brain injury
MCI: Mild cognitive impairment
FTD: Frontotemporal dementia
CSF: Cerebrospinal fluid
PET: Positron emission tomography
