# Supplementary material for: Plasma glial fibrillary acidic protein as a biomarker of disease progression in Parkinson’s disease: a prospective cohort study
Source: BMC Med. 2023 Nov 6;21:420. doi: 10.1186/s12916-023-03120-1 (PMC10626747; doi:10.1186/s12916-023-03120-1)
Supplement: Supplementary file 1 — Additional file 1: Table S1. Plasma GFAP levels of the included participants. Table S2. Correlation of plasma GFAP with other biomarkers at each visit. Table S3. Correlation of plasma GFAP with other biomarkers over time. Table S4. Comparison of clinical scores between PD patients with upward or downward trends of GFAP. Table S5. Cox proportional hazards regression models .Fig S1. Comparison of clinical progress between PD patients with upward or downward trends of GFAP. [file 12916_2023_3120_MOESM1_ESM.docx]

**Supplementary Online Content**

**Additional file 1: Table. S1. Plasma GFAP levels of the included participants**

**Additional file 1: Table. S2. Correlation of plasma GFAP with other biomarkers at each visit**

**Additional file 1: Table. S3. Correlation of plasma GFAP with other biomarkers over time**

**Additional file 1: Table. S4. Comparison of clinical scores between PD patients with upward or downward trends of GFAP**

**Additional file 1: Table. S5. Cox proportional hazards regression models**

**Additional file 1: Fig. S1. Comparison of clinical progress between PD patients with upward or downward trends of GFAP**

**Additional file 1: Table. S1.** **Plasma GFAP levels of the included participants**

|  | Sample size | Mean (SD),  pg/ml | Minimum, pg/ml | Maximum, pg/ml | p value |
| --- | --- | --- | --- | --- | --- |
| HC | 95 | 57.89 (23.54) | 17.47 | 177.77 |  |
| PD_baseline | 184 | 69.80 (36.18) | 24.85 | 258.61 | p<0.001* |
| PD_1 year | 184 | 72.32 (38.54) | 23.78 | 268.45 |  |
| PD_2 year | 124 | 74.12 (40.79) | 8.63 | 256.72 |  |

Abbreviations: GFAP, glial fibrillary acidic protein; HC, healthy control; PD, Parkinson’s disease.

*p value based on Friedman test which compares the change of GFAP levels of PD patients at baseline and at 1-year and 2-year follow-up.

**Additional file 1: Table. S2. Correlation of plasma GFAP with other biomarkers at each visit**

|  | **NfL** | **Aβ40** | **Aβ42** | **P-tau181** | **Aβ42/Aβ40** |
| --- | --- | --- | --- | --- | --- |
| **Baseline, coefficient** | 0.568** | 0.425** | 0.320** | 0.303** | 0.034 |
| **1-year, coefficient** | 0.570** | 0.560** | 0.475** | 0.355** | 0.066 |
| **2-year, coefficient** | 0.657** | 0.504** | 0.338** | 0.453** | -0.166 |

Abbreviations: GFAP, glial fibrillary acidic protein; NfL, neurofilament light chain; Aβ, amyloid-beta; p-tau181, phosphorylated tau-181.

P values were examined using Spearman correlation analyses. **p<0.01, *p<0.05.

**Additional file 1: Table. S3. Correlation of plasma GFAP with other biomarkers over time**

|  | Linear mixed-effects model |  |  |
| --- | --- | --- | --- |
|  | *β* (95% CI) | *P* value | FDR-corrected *P* value |
| Aβ40 | 0.660 (0.525 ~ 0.796) | <0.001* | 0.05* |
| Aβ42 | 6.637 (5.116 ~ 8.159) | <0.001* | 0.001* |
| NfL | 2.137 (1.776 ~ 2.497) | <0.001* | 0.001* |
| p-tau181 | 5.260 (3.071 ~ 7.449) | <0.001* | 0.001* |
| Aβ42/Aβ40 | 77.625 (-158.099 ~ 313.349) | 0.518 | 0.518 |

Abbreviations: GFAP, glial fibrillary acidic protein; NfL, neurofilament light chain; Aβ, amyloid-beta; p-tau181, phosphorylated tau-181.

*Significant based on linear mixed-effects models.

**Additional file 1: Table. S4.** **Comparison of clinical scores between PD patients with upward or downward trends of GFAP**

|  |  | Downward  (n = 85) | Upward  (n = 99) |
| --- | --- | --- | --- |
| UPDRS-I score, mean (SD) | Baseline | 1.08 (1.73) | 0.91 (1.46) |
|  | 1 year | 1.11 (1.48) | 1.23 (1.52) |
|  | 2 year | 1.43 (1.50) | 1.71 (1.72) |
| UPDRS-II score, mean (SD) | Baseline | 6.02 (3.95) | 5.82 (4.38) |
|  | 1 year | 7.52 (4.36) | 7.29 (4.25) |
|  | 2 year | 7.15 (4.74) | 7.97 (5.13) |
| UPDRS-III score, mean (SD) | Baseline | 22.45 (8.94) | 23.40 (8.61) |
|  | 1 year | 26.42 (8.93) | 27.25 (9.22) |
|  | 2 year | 29.67 (9.16) | 33.00 (9.06) |
| H&Y stage, mean (SD) | Baseline | 1.87 (0.39) | 1.91 (0.27) |
|  | 1 year | 2.05 (0.30) | 2.04 (0.30) |
|  | 2 year | 2.19 (0.40) | 2.21 (0.49) |
| MoCA score, mean (SD) | Baseline | 25.78 (3.31) | 25.18 (3.67) |
|  | 1 year | 25.00 (3.51) | 24.77 (3.80) |
|  | 2 year | 24.72 (4.17) | 24.03 (4.24) |
| FAB score, mean (SD) | Baseline | 16.15 (1.98) | 16.05 (2.43) |
|  | 1 year | 16.32 (2.01) | 15.95 (2.19) |
|  | 2 year | 15.92 (2.53) | 15.51 (2.40) |

Abbreviations: PD, Parkinson’s disease; GFAP, glial fibrillary acidic protein; UPDRS-I, Unified Parkinson’s Disease Rating Scale part I; UPDRS-II, Unified Parkinson’s Disease Rating Scale part II; UPDRS-III, Unified Parkinson’s Disease Rating Scale part III; H&Y, Hoehn & Yahr; MoCA, Montreal Cognitive Assessment; FAB, frontal assessment battery.

**Additional file 1: Table. S5. Cox proportional hazards regression models**

Abbreviations: UPDRS-III, Unified Parkinson’s Disease Rating Scale part III; GFAP, glial fibrillary acidic protein; MoCA, Montreal Cognitive Assessment.

|  | Unadjusted model |  | Adjusted model |  |
| --- | --- | --- | --- | --- |
| Variable | *HR* (95% CI) | *P* value | *HR* (95% CI) | *P* value |
| Sex | 0.716 (0.344 ~ 1.493) | 0.373 |  |  |
| Age | 1.047 (1.009 ~ 1.086) | 0.015* |  |  |
| UPDRS-III | 1.111 (1.067 ~ 1.156) | <0.001* | 1.107 (1.063 ~ 1.153) | <0.001* |
| GFAP | 1.010 (1.003 ~ 1.018) | 0.006* | 1.009 (1.001 ~ 1.017) | 0.033* |
| MOCA | 0.908 (0.829 ~ 0.995) | 0.039* |  |  |
| Disease duration | 1.300 (0.956 ~ 1.767) | 0.094 |  |  |

**Additional file 1: Fig. S1.** **Comparison of clinical progress between PD patients with upward or downward trends of GFAP**


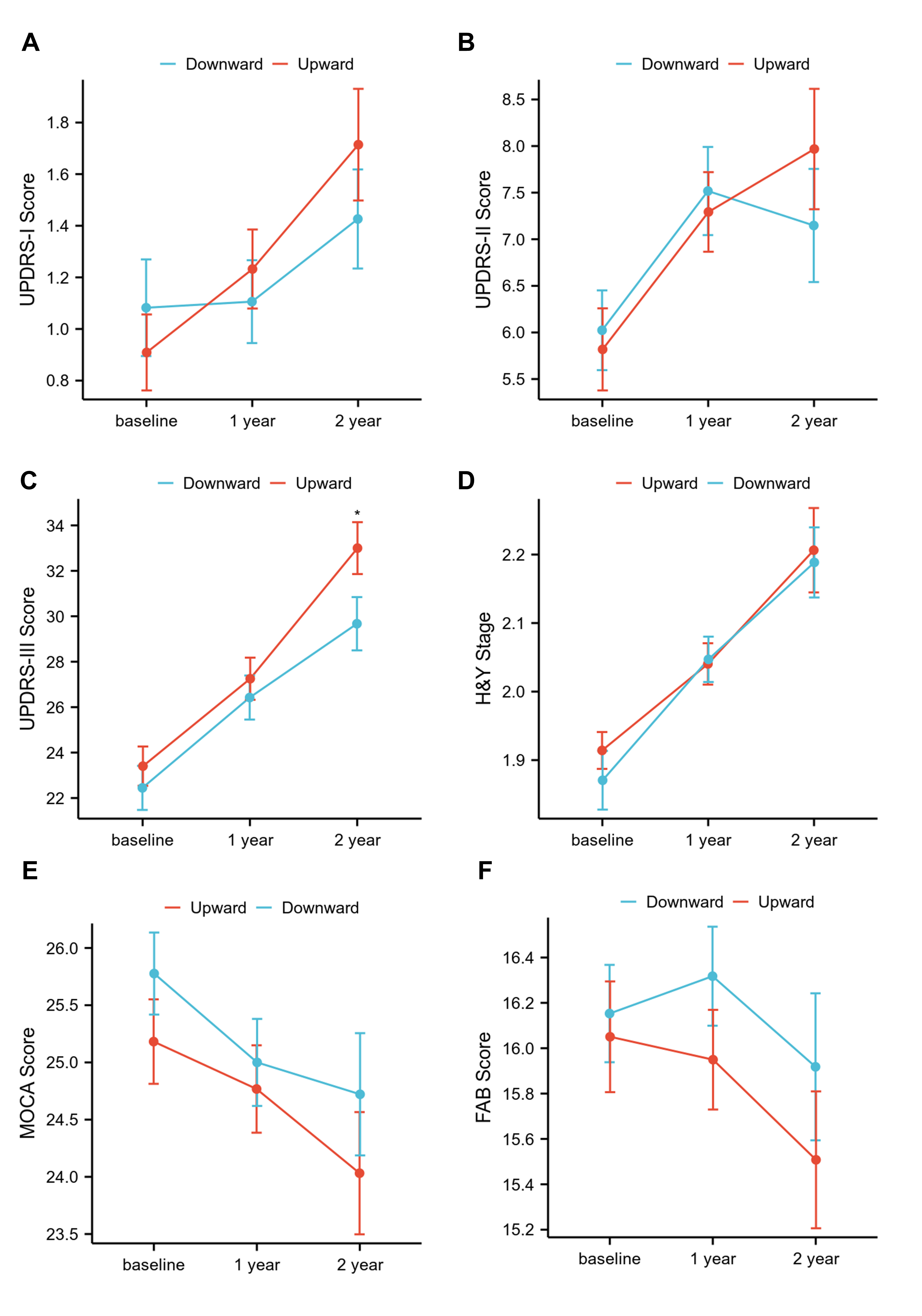


P values were examined using repetitive measurement deviation analyses.
